# Do Stress Responses Promote Leukemia Progression? An Animal Study Suggesting a Role for Epinephrine and Prostaglandin-E_2_ through Reduced NK Activity

**DOI:** 10.1371/journal.pone.0019246

**Published:** 2011-04-29

**Authors:** Shelly Inbar, Elad Neeman, Roi Avraham, Marganit Benish, Ella Rosenne, Shamgar Ben-Eliyahu

**Affiliations:** Neuroimmunology Research Unit, Department of Psychology, Tel Aviv University, Tel Aviv, Israel; Virginia Polytechnic Institute and State University, United States of America

## Abstract

In leukemia patients, stress and anxiety were suggested to predict poorer prognosis. Oncological patients experience ample physiological and psychological stress, potentially leading to increased secretion of stress factors, including epinephrine, corticosteroids, and prostaglandins. Here we tested whether environmental stress and these stress factors impact survival of leukemia-challenged rats, and studied mediating mechanisms. F344 rats were administered with a miniscule dose of 60 CRNK-16 leukemia cells, and were subjected to intermittent forced swim stress or to administration of physiologically relevant doses of epinephrine, prostaglandin-E_2_ or corticosterone. Stress and each stress factor, and/or their combinations, doubled mortality rates when acutely applied simultaneously with, or two or six days after tumor challenge. Acute administration of the β-adrenergic blocker nadolol diminished the effects of environmental stress, without affecting baseline survival rates. Prolonged β-adrenergic blockade or COX inhibition (using etodolac) also increased baseline survival rates, possibly by blocking tumor-related or normal levels of catecholamines and prostaglandins. Searching for mediating mechanisms, we found that each of the stress factors transiently suppressed NK activity against CRNK-16 and YAC-1 lines on a per NK basis. In contrast, the direct effects of stress factors on CRNK-16 proliferation, vitality, and VEGF secretion could not explain or even contradicted the *in vivo* survival findings. Overall, it seems that environmental stress, epinephrine, and prostaglandins promote leukemia progression in rats, potentially through suppressing cell mediated immunity. Thus, patients with hematological malignancies, which often exhibit diminished NK activity, may benefit from extended β-blockade and COX inhibition.

## Introduction

A considerable body of evidence implicates physiological stress responses as modulators of the progression of several malignancies, including tumors of the breast [1], [2], [3], skin [4], [5], reproductive system [6], [7], and gastrointestinal tract [8], [9]. Studies in tumor-bearing patients suggest that cancer diagnosis and its treatment are associated with substantial psychological and physiological distress [10], which were reported to predict accelerated disease progression [11], [12]. Inversely, factors such as social support and optimism were suggested to predict prolonged survival in cancer patients [13], further hinting at a modulating impact of stress responses and stress hormones in cancer progression.

While human studies are clearly limited in their ability to delineate specific mechanisms mediating these alleged effects of distress, animal studies have causally linked specific neuro-endocrine stress responses to accelerated cancer progression [3], [4], [5], [14], [15], [16]. Activation of the sympathetic nervous system (SNS) and the hypothalamic–pituitary–adrenal (HPA) axis was implicated as facilitating cancer progression, as were pro-inflammatory factors and prostaglandins [3], [14], [17], [18]. The mechanisms through which these stress factors can affect tumor progression are various. Studies have associated stress responses with suppression of cell-mediated immunity (CMI). This suppression includes reduced numbers of circulating CTL and NK cells, as well as reduced NK activity [14], [19] and reduced production of type-1 cytokines [20], [21]. More recently, catecholamines and prostaglandins were shown to directly influence specific tumor lines, promoting their progression through several cellular mechanisms. These include improved tumor cell invasion, migration, proliferation [22], [23], and the tumor release of pro-angiogenic factors such as vascular endothelial growth factor (VEGF) [24], as well as through decreased tumor anoikis (i.e., increased tumor cell survival after separation from the extracellular matrix) [7]. Irrespective of whether stress hormones promote cancer progression directly by affecting malignant cells, or indirectly by suppressing host immunity or altering other aspects of host physiology, it appears that stress hormones and pro-inflammatory factors play a substantial role in the progression of solid tumors.

To date, these issues have not been directly addressed in the context of leukemia; nonetheless, they may well be relevant. Indeed, studies have shown that poorer prognosis in leukemia patients is associated with suppressed CMI responses [25], [26], [27], [28], and with alteration in the angiogenic/invasive profile [29], [30], [31]. Accordingly, levels of autologous NK activity were found to predict survival in leukemia patients [25], [26], and graft-versus-leukemia studies strongly suggest that an efficient NK response after bone marrow transplantation can control acute leukemia [32]. Over-expression of angiogenic/invasive factors, including VEGF and MMP9, was found in patients with chronic lymphocytic leukemia (CLL), and microvessel count in tumor microenvironment was positively correlated to the clinical stage of these patients [29], [30], [31].

Patients undergoing treatment for leukemia were reported to exhibit high levels of anxiety and distress [33], [34], [35], and several psychosocial aspects were implicated as prognostic factors in leukemia, including quality-of-life, depression, and anxiety [36],[37]. However, it remains unclear whether patients' stress responses can be causally implicated in accelerating leukemia progression or in increased mortality. Clinical and preclinical studies addressing this question at the mechanistic or the therapeutic level are scarce.

To address these issues in an animal model, in the current study we used the CRNK-16 leukemia line which is syngeneic to the F344 rat. This line originated from a naturally occurring leukemia that is highly malignant and is the major cause of death in aged F344 rats [38]. CRNK-16 cells were reported to form metastases in the omentum, lymph nodes, spleen, liver, thymus and lungs [38]. Our more recent work also established that administration of a minuscule dose of 60 CRNK-16 cells is sufficient to induce significant levels of mortality, and that this tumor expresses low levels of MHC-I and is susceptible *in vivo* to lysis by NK cells, but apparently not by adaptive immunity [39]. Thus, the CRNK-16 cell line provides an opportunity to study the effects of stress hormones on leukemia progression *in vivo* in a biologically relevant setting with potential clinical significance.

Hence, in the following studies, using this tumor line and assessing survival rates in F344 rats, we tested (i) the effects of administration of apparently biologically relevant doses of epinephrine, corticosterone, and prostaglandin E_2_ (PGE_2_), (ii) the impact of subjecting rats to stressful environmental conditions, and (iii) the use of a β-blocker and a non-selective COX inhibitor in stress and non-stress conditions. We also studied the *in vivo* impact of these hormones (and that of CRNK-16 cells themselves) on NK activity, as well as their *in vitro* direct effects on CRNK-16 proliferation, vitality, and VEGF secretion.

## Materials and Methods

### Animals

Male and female Fischer 344 rats (Harlan Laboratories, Jerusalem) were housed 4 per cage, with saw-dust bedding, under a 12∶12 hours light/dark cycle at 22±1°C, with free access to standard food and fresh water. Animals were acclimatized to the vivarium for at least 4 weeks, and were 12 to 16 weeks old at the beginning of experimentation (in any given experiment, all animals were of the same age), average weight was 260 g for males and 175 g for females. All experiments were approved by the Institutional Animal Care and Use Committee of Tel Aviv University (permit number p-07-004), and all efforts were made to minimize animal suffering.

### General experimental procedure

Rats were handled on 3 consecutive days before each experiment to reduce unwanted procedural stress. Gender and the order of stress initiation, drug administration, and tumor injection, were all counterbalanced across groups.

### CRNK-16 tumor cells and their *in vivo* administration

The CRNK-16 cell line was kindly provided by Dr. W.H. Chambers, from the University of Pittsburgh Cancer Institute. Morphologically, these leukemia cells resemble large granular lymphocytes (LGL), and are NKRP-1 (CD161) and CD45 positive, and CD3 negative [40]. Also, these cells constitutively express the NK activation marker – gp42 [41], and the Ly49, CD94 and NKG2-A/C receptors [42], [43]. Cells were maintained in complete medium (CM) (RPMI 1640 supplemented with 10% heat-inactivated FCS, 50 µg/ml gentamicin, 2 mM L-glutamine, 0.1 mM non-essential amino acids and 1 mM sodium pyruvate (purchased from Biological Industries, Beit Haemeq, Israel)) at 100% humidity, 5% CO_2_ at 37°C. CRNK-16 cells were administered i.v. under light halothane anesthesia at a dose of 60 cells per rat suspended in 1 ml of PBS with 0.1% bovine serum albumin.

### Measurement of survival

In all experiments, rats were inspected for signs of morbidity once or twice daily for a period of 80 days following tumor injection. Animals were defined as morbid given two or more of the following criteria: loss of more than 10% of body weight, apathy, dehydration, red circles around the eyes, overt inactivity, or paralysis. Based on our experience, these symptoms characterize a progressive phase of disseminated tumors, causing death within approximately 24–48 hours. Animals defined as morbid were euthanized and inspected to verify the development of metastases. Tumor masses were established in various body organs, including kidneys, bones, lymph nodes, lungs, liver and colon. These animals were included in the mortality report, and considered to have died on the next day. Animals that survived the 80-day period were euthanized and inspected for tumor masses or irregularity in internal organs (specifically spleen, liver, kidney, and all organs in the chest cavity) and for bone softness at this time point. None of the survivors showed any signs of malignancy, while all rats included in the mortality report exhibited one or more signs of tumor development, and most had solid metastases. In our previous study using this tumor model [39], we inspected the surviving animals for an additional 3-month period and recorded only 2 cases of mortality within more than 200 animals. Thus, rats that survived this period are unlikely to later show CRNK-16 related morbidity.

### Environmental swim stress

A 5-gram weight was attached to the tails of stressed rats. Each stressed rat was then placed for 3 minutes in a tank containing water 35 centimeters deep, at 24°C, followed by a 3-minute rest period. This procedure was repeated 5 times successively. Environmental swim stress was counterbalanced across cages (not within cage) in order to avoid unwanted stress in controls.

### Materials

#### Prostaglandin E_2_ (PGE_2_; Sigma, Israel)

In the survival and NK activity studies (Exp. 1–5), PGE_2_ was first dissolved in ethanol and then diluted in phosphate buffered saline (PBS), reaching a final concentration of less than 10% ethanol in the preparation. In order to maintain a relatively constant absorption level, PGE_2_ was administered subcutaneously (0.8 mg/kg/ml) in a slowly absorbed emulsion (consisting of 4 parts of the PBS/ethanol preparation containing the active ingredient, 3 parts of mineral oil, and 1 part of mannide-monooleate – a non specific surface active emulsifier – all purchased from Sigma, Israel). Based on our experience, using various compounds, this emulsion prolongs the absorption of its contents to at least several hours, which is essential when compounds with a short half-life, such as PGE_2_ and epinephrine, are used. Control rats were administered with the exact same emulsion excluding the PGE_2_. As PGE_2_ acts locally in a paracrine/autocrine manner [44], the relevancy of its systemic serum level is unclear. Nevertheless, this dose, administered in the slowly absorbed emulsion, is apparently physiologically relevant, as its effects on several immune indices, are equivalent to those of surgical stress, and can be blocked by doses of COX inhibitors similar to those needed to block physiological effects after surgery [3], [17], [19]. For the *in vitro* experiments (Exp. 6–7), PGE_2_ was dissolved and diluted to a concentration of 1.43×10^−3^ M in 20% ethanol and 80% PBS, and further diluted in CM to the final concentrations (10^−6^ M to 10^−8^ M).

#### Epinephrine (Sigma, Israel)

In the survival and NK activity studies, epinephrine was administered subcutaneously (0.5 mg/kg) in the aforementioned slowly absorbed emulsion. Epinephrine was first dissolved in the PBS fraction of the emulsion. This dose, administered in the slowly absorbed emulsion, is apparently physiologically relevant, as its effects on several immune indices are equivalent to those of environmental swim stress and surgical stress, and can be blocked with the same doses of β-blockers used to block the effects of these environmental stressors (unpublished data from our laboratory). Control rats were administered with the exact same emulsion excluding the epinephrine. For the *in vitro* experiments, epinephrine was dissolved and diluted in CM to the final concentrations (10^−5^ M to 10^−7^ M).

#### Corticosterone (Sigma, Israel)

In the survival and NK activity studies, corticosterone was dissolved overnight in corn oil and administered subcutaneously at a dose of 3 mg/kg, twice, 5 hours apart. This dose induces physiological high levels of plasma corticosterone (500–800 ng/ml) for 4–5 hours per injection [45], which are similar to levels reached after exposure to environmental stressors [14]. Control rats were administered twice with corn oil at the same time points. For the *in vitro* experiments, corticosterone was dissolved in ethanol to a concentration of 0.03 M and further diluted in CM to the final concentrations (10^−6^ M to 10^−8^ M).

**Nadolol** (Sigma, Israel), a hydrophilic nonselective β-adrenergic antagonist, which does not cross the blood-brain-barrier. In Exp. 3, nadolol was dissolved in PBS and injected twice subcutaneously: 10 minutes prior to environmental swim stress, at a dose of 0.4 mg/kg, and again 60 minutes after environmental swim stress, i.e., with tumor injection, at a dose of 0.2 mg/kg. In Exp. 4, the second injection was administered in the aforementioned slowly absorbed emulsion at a dose of 4.5 mg/kg nadolol. Control rats were administered twice with the appropriate vehicles at the same time points. For the *in vitro* experiments, nadolol was dissolved and diluted in CM to the final concentrations (10^−5^ M to 10^−7^ M).

**Indomethacin** (Sigma, Israel), a nonselective COX inhibitor, with a slight preference to COX-1 inhibition, and a half-life of about 4 hours in rats [46]. Indomethacin was dissolved in propylene glycol and administered subcutaneously (4 mg/kg, 2 ml/kg). Control rats were administered with the propylene glycol.

**Metaproterenol** (Sigma, Israel), a moderately selective β_2_-adrenergic receptor agonist, was dissolved and diluted in CM to the final concentrations (10^−5^ M to 10^−7^ M).

### Flow Cytometry of NK and CRNK-16 cells

FACS analysis was used to assess the concentration of granulocytes, lymphocytes, T, NKT, and NK cells in the blood (Exp. 5) and to count CRNK-16 cells and determine their vitality (Exp. 7). Lymphocytes and granulocytes were identified according to their forward and side scatters. As described previously by Chambers et al. [47], NK cells were identified as being above a level of fluorescence intensity that distinguishes between bright and dim populations of CD161 positive cells. This previous study also demonstrated that CD161 is expressed by 94% of blood LGL cells of the rat, and that the NK cytolytic activity was totally contained in the CD161^bright^ cell population. Thus, NK cells were herein identified as CD161^bright^ lymphocytes, based on FITC-conjugated anti-CD161 labeling and scatter properties [48]. T cells were identified as CD5^+^CD161^−^ and NKT cells as CD5^+^CD161^+^, based on PE-conjugated anti-CD5 labeling and scatter properties. CRNK-16 cells were studied in a separate experiment, and were the only cell type in the FACS preparation. To assess the total numbers of each specific subset per microliter of blood, (Exp. 5) and the number of CRNK-16 cells per well (Exp. 7), we added 20 µm polystyrene microbeads (Duke Scientific, Palo Alto, CA) at a concentration of 600/µl of sample studied. Following cytometry, the formula: CD161^bright^×microbeads/600 was used to calculate NK cell concentrations and similar formulas were used for each other subset of leukocytes (and for CRNK-16 cells). The coefficient of variation for this method was found in our laboratory to be 6% for identical samples. In order to determine CRNK-16 vitality, cells were stained with 7-amino-actinomycin D (7-AAD), a ready-to-use solution for the exclusion of nonviable cells in flow cytometric analysis (for details see Schmid et al. [49]).

### Assessment of blood NK Activity (cytotoxicity)

#### Preparation of blood effector cells

Rats were anesthetized with isoflurane, and 1 ml of blood was withdrawn by cardiac puncture into syringes containing 30 units of heparin. Whole blood was then washed once with PBS and twice with CM. This method was used to remove sera while maintaining the original composition of leukocytes in the blood.

#### Preparation of target cells

CRNK-16 and YAC-1 [50] cells were washed with CM. Both CRNK-16 cells and YAC-1 cells (5×10^6^ of each) were incubated for 1 h with 100 µCi ^51^Cr (Danyel Biotech, Rehovot, Israel) in 100 µl saline, 100 µl heat-inactivated fetal calf serum (FCS), and 75 µl CM. Following incubation, cells were washed three times in CM (300×g for 10 minutes) and adjusted to a concentration of 5×10^4^/ml.

#### NK activity (cytotoxicity) assay

The standard whole-blood 4 hour ^51^Cr release assay was used to assess leukocyte anti-tumor cytotoxicity against the CRNK-16 syngeneic line and the standard YAC-1 line. This procedure was previously described elsewhere [19]. To create four different effector-to-target (E∶T) ratios, blood effector cells were serially diluted (1∶2) beginning at 1/4 of their original concentration in the blood, and co-incubated with a fixed number of target cells. Specifically, aliquots of 150 µl of effector cell suspension (from washed blood) were placed in wells of a microtiter plate and serially diluted to create the 4 E∶T ratios. 5000 labeled target cells (CRNK-16 or YAC-1) in 100 µl complete media were then added to each well. Plates were centrifuged (400 g×10 min) and incubated at 37°C, 100% humidity, for 4 hours. Plates were then centrifuged again and 100 µl of supernatant was removed from each well and counted in a gamma counter to determine experimental ^51^Cr release. Spontaneous release was obtained from wells containing target cells and CM only, and total release was obtained from wells containing 1% Triton X-100. Percent cytotoxicity was calculated by the following formula: Percent Cytotoxicity = 100×[(experimental release minus spontaneous release)/(total release minus spontaneous release)]. Our previous studies [51], [52] indicate that cytotoxicity in this assay depends on NK cells, since their selective depletion nullified all specific cytotoxicity.

#### Assessment of Cytotoxicity per NK Cell

Since we recorded the number of NK cells in each sample tested for cytotoxicity, we were able to mathematically align samples' NK cell concentration, according to the FACS analysis, to achieve common NK:CRNK-16 or NK:YAC-1 ratios, thereby assessing NK cytotoxicity on a per NK cell basis.

### *In vitro* ELISA assessment of supernatant VEGF levels

Standard R&D (Minneapolis, MN, USA) ELISA kits were used according to manufacturer's instructions.

### *In vitro* EIA assessment of serum PGE_2_ levels

Standard Cayman Chemicals (Ann Arbor, MI, USA) EIA kits were used according to manufacturer's instructions.

### Radio-Immuno-Assay for serum Corticosterone levels

A standard ^125^I double-antibody RIA kit (MP Biomedicals, Solon, OH, USA) was used according to the manufacturer's instructions.

### Statistical analysis

For statistical analysis of the survival experiments (Exp. 1–4), the Kaplan-Meier model of survival was used, followed by the Tarone-Ware test for pair-wise group comparisons. For experiments 5, 6 and 7, one- or two-way analyses of variance (ANOVA) were used to analyze percent cytotoxicity (NK activity), VEGF levels, and cell numbers and vitality. Provided significant group differences were indicated by ANOVA for experiment 5, Fisher's Protected Least Significant Differences (PLSD) contrasts were used to test specific pair-wise differences with respect to our hypothesized effects: PGE_2_, epinephrine and corticosterone will reduce NK activity compared to controls. Provided significant group differences were indicated by ANOVA for experiments 6–7, Scheffe' post hoc contrasts were used. For all experiments, p values of less than 0.05 were considered significant, and p values of 0.05–0.1 were considered marginally significant.

## Results

### Experiment 1: Epinephrine and PGE_2_, but not corticosterone, significantly reduced survival rates

Epinephrine (0.5 mg/kg s.c. in a slowly absorbed emulsion), PGE_2_ (0.8 mg/kg s.c. in a slowly absorbed emulsion) and corticosterone, (2 injections of 3 mg/kg s.c., 5 hours apart) or their respective vehicles (slowly absorbed emulsion or corn oil) were each administered simultaneously with tumor injection.

#### Experiment 1a: effects of PGE_2_ administration on survival

**Figure 1:**
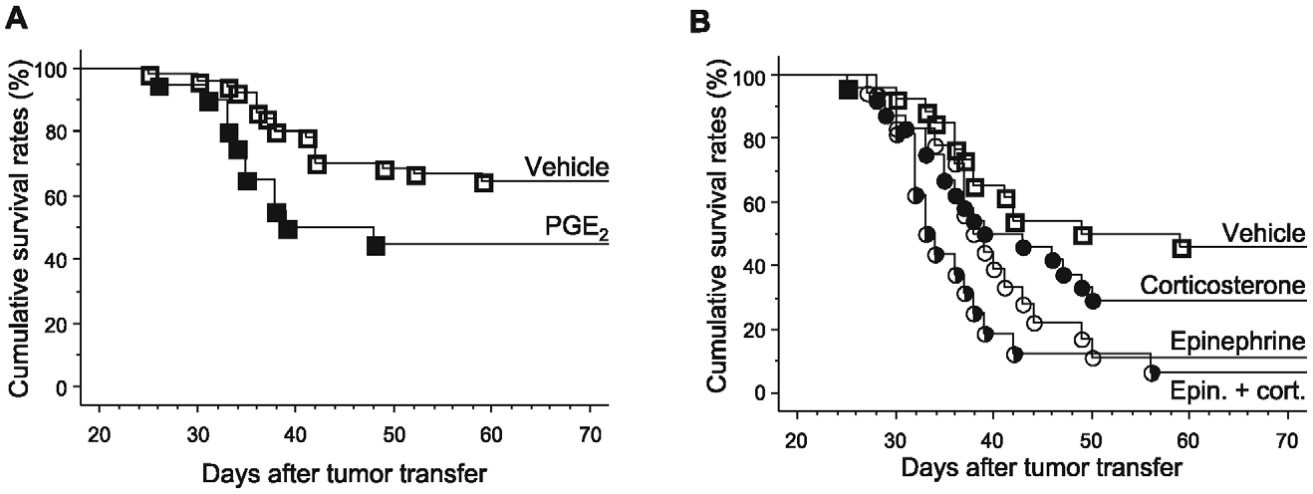
Effects of PGE_2_, epinephrine and corticosterone administration on survival rates. (A) PGE_2_ (▪, n = 20), administered simultaneously with tumor injection, significantly decreased survival rates compared to vehicle (□, n = 25) administration (control). (B) Epinephrine, alone (○, n = 18) or in combination with corticosterone (half-filled circles, n = 16), significantly decreased survival rates, compared to vehicle control treatment (□, n = 26), while corticosterone (•, n = 24) did not cause a significant effect.

Female rats were injected with either PGE_2_ (n = 20) or vehicle (n = 25). As indicated in Fig. 1A, PGE_2_ administration significantly decreased survival rates (Kaplan-Meier model, Tarone-Ware, p<0.05).

#### Experiment 1b: effects of epinephrine and corticosterone administration on survival

Male rats were injected with either epinephrine (n = 18), corticosterone (n = 24), a combination of the two (n = 16), or vehicle (n = 26). As indicated in Fig. 1B, epinephrine alone, or co-administered with corticosterone, significantly decreased survival rates as compared to vehicle-treated rats (Kaplan-Meier model, Tarone-Ware, p<0.05 and p<0.05, respectively). Corticosterone alone did not significantly alter survival rates.

### Experiment 2: Co-administration of epinephrine and corticosterone decreases survival rates when given simultaneously with tumor transfer or 2 or 6 days later

**Figure 2:**
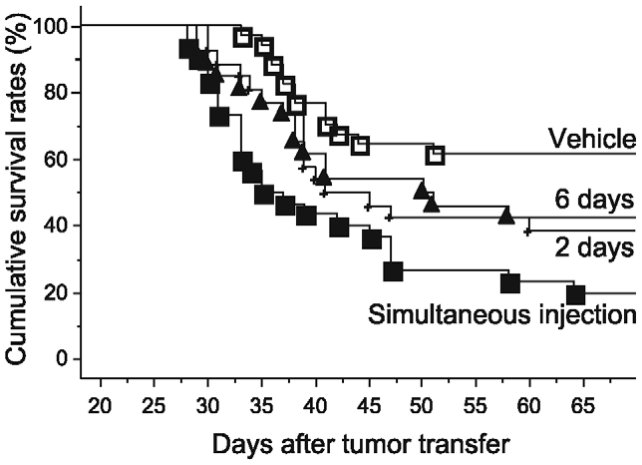
Time dependent effects of co-administration of epinephrine and corticosterone on survival rates. Co-administration of epinephrine and corticosterone simultaneously with tumor injection (▪, n = 30) significantly decreased survival rates. Administration of the same drug combination, 2 (+, n = 26) or 6 (▴, n = 26) days after tumor injection, led to a smaller decrease in survival rates, becoming significantly lower than control (□, n = 34) when these groups were combined.

Male and female rats were co-administered with epinephrine (0.5 mg/kg s.c. in a slowly absorbed emulsion) and corticosterone (3 mg/kg s.c.; twice, 5 hours apart), simultaneously with (n = 30), 2 days (n = 26) or 6 days (n = 26) after tumor injection, or administered with the relevant vehicles at these time points (total of 34 rats). As indicated in Fig. 2, co-administration of epinephrine and corticosterone significantly decreased survival rates when injected simultaneously with tumor cells (Kaplan-Meier model, Tarone-Ware, p<0.05), but less so when injected 2 or 6 days thereafter (Kaplan-Meier model, Tarone-Ware, p = 0.066, p<0.11, respectively), as compared to vehicle-treated rats. When the two groups of 2 and 6 days were combined into one “delayed drug administration” group, a significant difference, compared to the vehicle control group, was evident (Kaplan-Meier model, Tarone-Ware, p<0.05).

### Experiment 3: Environmental swim stress reduces survival rates, and the β-adrenergic antagonist, nadolol, blocks this effect

**Figure 3:**
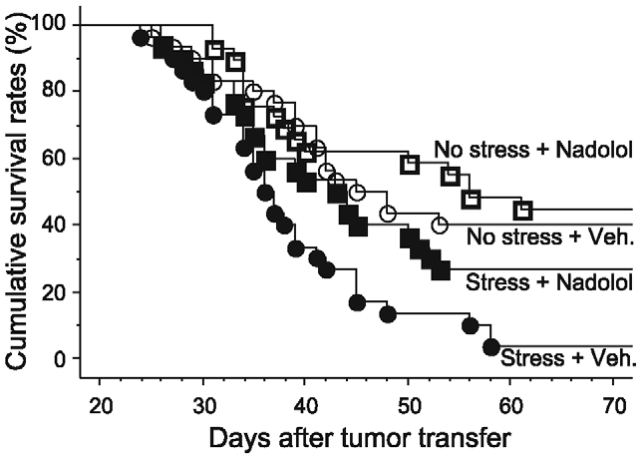
Effects of environmental swim stress and nadolol treatment on survival of leukemic rats. Survival rates of non-stressed rats, treated with an acute administration of nadolol (□, n = 29) or vehicle (○, n = 30) were compared to those of stressed rats, treated with nadolol (▪, n = 29) or vehicle (•, n = 30). Environmental swim stress significantly decreased survival rates. This effect was blocked by nadolol, which did not significantly affect the survival of non-stressed rats.

To ascertain that physiological levels of stress hormones released endogenously under stressful conditions can affect survival rates, the following study was conducted. Male rats were either subjected to environmental swim stress, or served as controls and remained undisturbed in their home cages. In each group, rats were either treated with the non-specific β-blocker, nadolol, or injected with saline (n = 29–30/group). Nadolol was subcutaneously injected at 0.4 mg/kg, 10 minutes prior to the beginning of the environmental swim stress procedure, followed by 0.2 mg/kg, 1 hour after the termination of the procedure, at the time of tumor injection. PGE_2_ serum levels were also measured in stressed and non stressed rats. As indicated in Fig. 3, in saline-treated rats, environmental swim stress significantly decreased survival rates (Kaplan-Meier model, Tarone-Ware, p<0.05) compared to non-stressed controls. Nadolol, which had no effect on survival of non-stressed rats, significantly blocked the deleterious effect of environmental swim stress on survival (p<0.05). PGE_2_ serum levels were unaffected by environmental swim stress compared to controls.

### Experiment 4: Improving survival rates by targeting tumor-associated or baseline levels of catecholamines and prostaglandins

**Figure 4:**
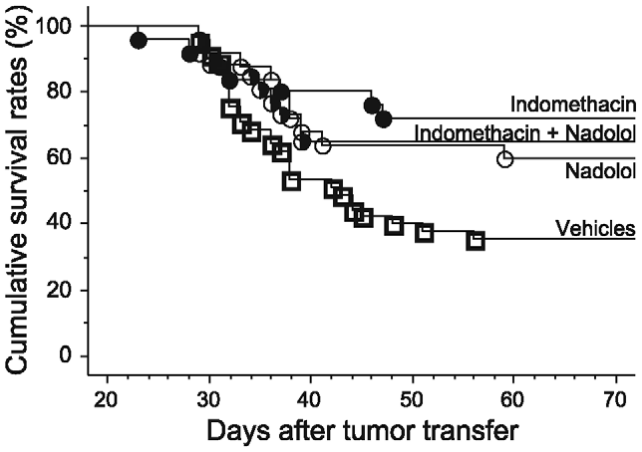
Effects of targeting tumor-associated and/or baseline levels of prostaglandins and catecholamines in CRNK-16-bearing rats. Indomethacin, with (half-filled circles, n = 26) or without (•, n = 25) a prolonged administration of nadolol, significantly improved survival rates. Nadolol alone (○, n = 25) marginally improved survival (p = 0.058), compared to vehicle (□, n = 45) control treatment.

Given that epinephrine and PGE_2_ decreased survival rates, we herein tested whether endogenous levels of these hormones, which are not induced by environmental stressful conditions, can also impact survival rates. The mere *in vivo* presence of leukemia may increase plasma levels of stress hormones and PGE_2_. Many tumors were shown to secrete PGE_2_ [53], [54], [55], presumably to suppress anti-tumor immunity, and the general stress response of the host toward a disease includes various stress-related factors [56]. Therefore, we tested whether antagonizing tumor-associated or baseline levels of prostaglandins or catecholamines for 24–48 hours following tumor challenge can improve survival rates. Specifically, one hour before tumor injection, male rats were administered with either: (i) nadolol (an acute loading dose of 0.4 mg/kg s.c. dissolved in saline, followed by an additional 4.5 mg/kg maintenance dose in a slowly absorbed emulsion given with tumor injection; n = 25), (ii) indomethacin (4 mg/kg i.p.; n = 25), (iii) a combination of (i) and (ii) (n = 26), or (iv) their vehicles (n = 45). As indicated in Fig. 4, indomethacin significantly increased survival rates, both alone (Kaplan-Meier model, Tarone-Ware, p<0.05) and in combination with nadolol (Tarone-Ware, p<0.05), whereas nadolol alone only marginally improved survival rates (Kaplan-Meier model, Tarone-Ware, p<0.058), compared to vehicle-treated rats.

### Experiment 5: *In vivo* administration of PGE2, epinephrine, or corticosterone reduces NK activity without significantly affecting NK cell numbers

**Figure 5:**
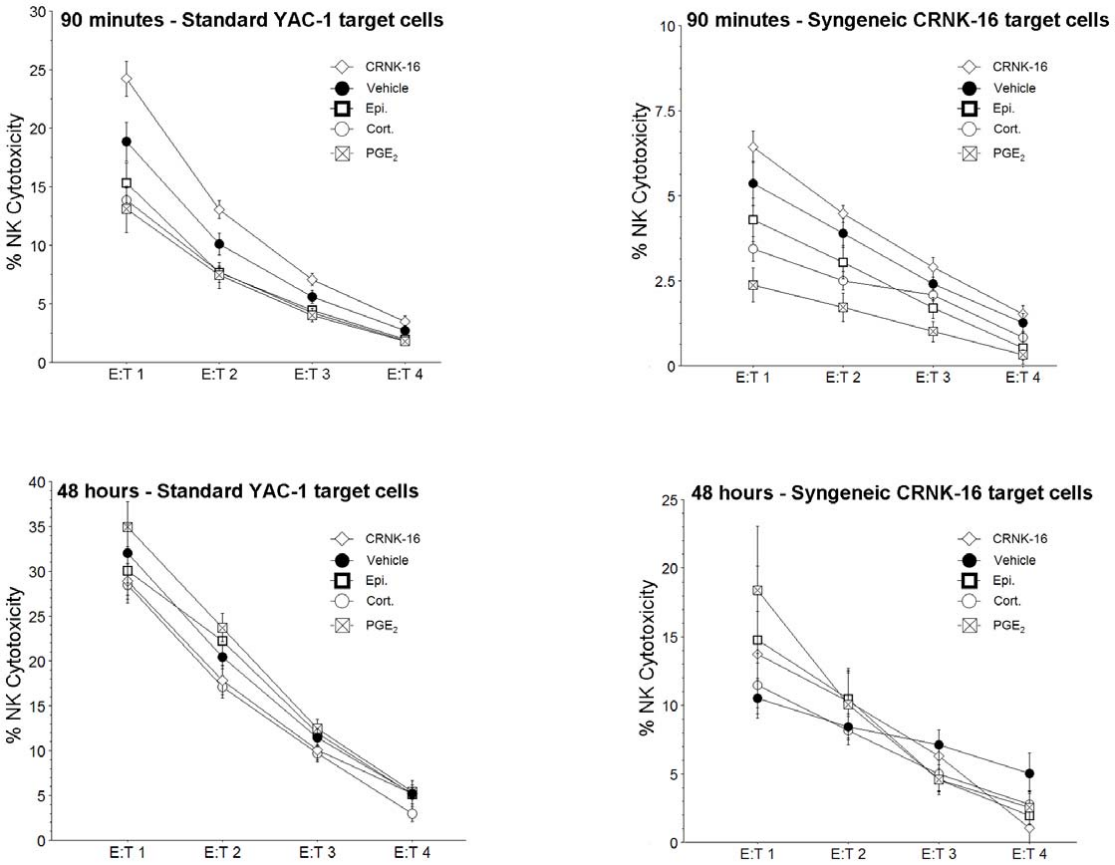
Effects of administration of epinephrine, corticosterone, PGE_2_ and CRNK-16 cells on NK activity. Ninety minutes (but not 48 hours) after their administration, epinephrine, corticosterone and PGE_2_, each significantly decreased NK activity against both the standard YAC-1 and the syngeneic CRNK-16 lines. Administration of CRNK-16 cells (n = 10/time point) increased NK activity against YAC-1 line, compared to vehicle control treatment at 90 minutes, but not 48 hours. Data are presented as mean+SEM (n = 10/group/time point).

Male rats were injected with epinephrine, corticosterone, PGE_2_, 60 CRNK-16 cells or vehicle (n = 8–10 per group). Ninety minutes or 48 hours later, blood was withdrawn by cardiac puncture from all rats and assayed for NK activity against the syngeneic CRNK-16 and the standard YAC-1 line (see methods). Serum levels of PGE_2_ and corticosterone were also measured. As indicated in Fig. 5, 90 minutes after their administration, corticosterone, epinephrine and PGE_2_, each significantly reduced NK activity on a per NK level (p<0.05), against both tumor lines, while CRNK-16 cell administration significantly increased NK activity against the YAC-1 line. Forty-eight hours after administration, no differences in NK activity were found between groups against both lines.

NK cell numbers were not significantly affected by the treatments, although other cell types (e.g., T and NKT cells) were significantly changed at 90 minutes (See Table S1), but not at 48 hours (See Table S2).

Ninety minutes after drug/tumor injection, corticosterone serum levels were significantly increased in the epinephrine and PGE_2_ groups, and significantly decreased in the CRNK-16 group (See Fig. S1). Administration of PGE_2_ significantly elevated PGE_2_ serum levels to maximum detection levels (∼2000 pg/ml) at 90 minutes, which returned to baseline levels at 48 hours. These levels were unaffected by other treatments, compared to vehicle (See Fig. S2).

### Experiment 6: CRNK-16 does not secrete VEGF, spontaneously or in the context of stress hormones or PGE_2_

#### Experiment 6a: CRNK-16 cells exhibit undetectable levels of VEGF secretion

**Figure 6:**
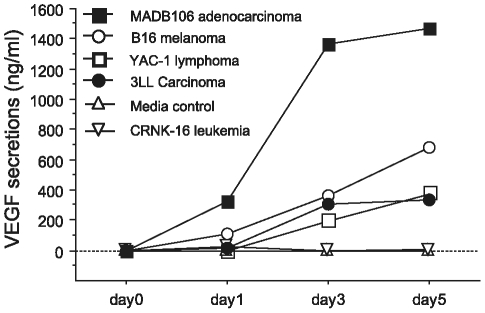
Spontaneous secretion of VEGF by different malignant cell lines. YAC-1 T-cell lymphoma, B16 melanoma, 3LL Lewis lung carcinoma, and the MADB106 mammary adenocarcinoma cells served as positive controls and secreted increasing amounts of VEGF on days 1, 3 and 5, while CRNK-16 showed undetectable levels.

CRNK-16 cells were washed in CM and incubated in 24-well flat-bottomed plates (500,000 cells in 1 ml CM per well). YAC-1 T-cell lymphoma [50], B16 melanoma [57], 3LL Lewis lung carcinoma [58], and the MADB106 mammary adenocarcinoma [59] cell lines were also incubated under the same conditions and served as positive controls. The control condition included CM without tumor cells. The plates were incubated at 100% humidity, 37°C, 5% CO_2_. Supernatant fluid was harvested after incubation of 1, 3 and 5 days, and assayed for murine VEGF using ELISA kits (R&D, Minneapolis, MN, USA). All cell lines spontaneously secreted VEGF, while the CRNK-16 line showed undetectable VEGF secretion levels at all times (see Fig. 6).

#### Experiment 6b: No impact of PGE_2_, corticosterone, and catecholamine associated drugs on VEGF secretion by CRNK-16

Some tumor lines were reported to increase their VEGF secretion when incubated for 3 or 6 hours with catecholamines [60]. Although CRNK-16 cells did not spontaneously secrete detectable levels of VEGF, we tested whether various drugs could induce such secretion. CRNK-16 cells were washed in CM and incubated in 24-well flat-bottomed plates with our standard CM with or without fetal calf serum, for 3 or 6 hrs (500,000 cells in 1 ml CM per well). Additionally, wells were supplemented with either: (i) norepinephrine (10^−5^ M/10^−6^ M/10^−7^ M); (ii) metaproterenol (10^−5^ M/10^−6^ M/10^−7^ M); (iii) corticosterone (10^−6^ M/10^−7^ M/10^−8^ M); (iv) prostaglandin E_2_ (10^−6^ M/10^−7^ M/10^−8^ M); (v) nadolol (10^−5^ M/10^−6^ M/10^−7^ M); (vi) ethanol (10^−6^ M/10^−7^ M/10^−8^ M) which served as vehicle control for PGE_2_ and corticosterone (vii) CM control without any drugs. None of the hormones and drugs listed above induced VEGF production in the CRNK-16 cell line (data not shown).

### Experiment 7: Corticosterone and PGE_2_, but not epinephrine or metaproterenol, decreased CRNK-16 proliferation and vitality *in vitro*

**Figure 7:**
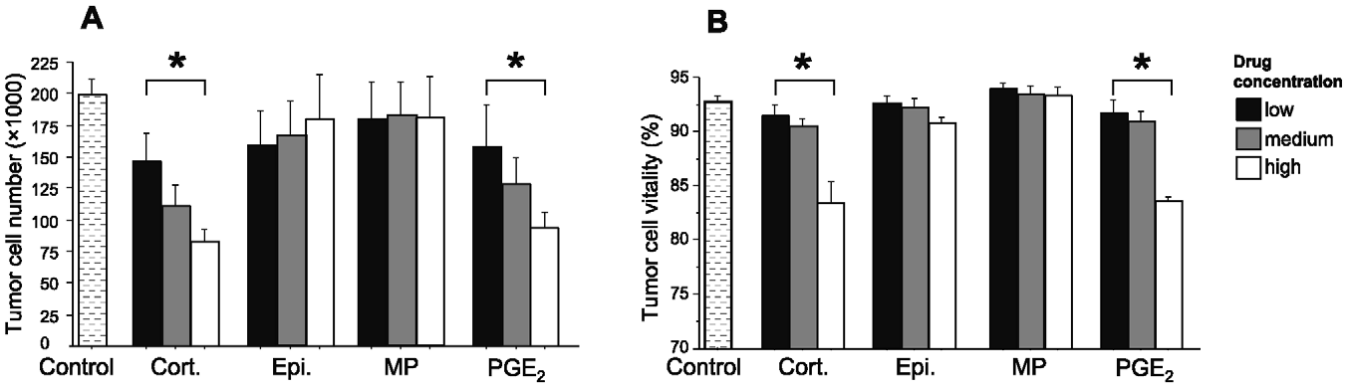
*In vitro* effects of stress hormones and PGE_2_ on CRNK-16 proliferation and vitality at 48 hours. Corticosterone and PGE2 reduced CRNK-16 cell proliferation and vitality in a dose dependent manner at 48 h. Epinephrine and metaproterenol did not affect proliferation (A) and vitality (B) rates. Data are presented as mean+SEM. * indicates a significant difference from the control group. Drug concentrations ranged from 10^−8^ M to 10^−5^ M, see Exp. 7 for details.

CRNK-16 cells were washed in CM and incubated in a 96-well flat-bottomed plate (30,000 cells in 200 µl CM per well) with either: (i) epinephrine (10^−5^ M/10^−6^ M/10^−7^ M); (ii) metaproterenol (10^−5^ M/10^−6^ M/10^−7^ M); (iii) corticosterone (10^−6^ M/10^−7^ M/10^−8^ M); (iv) PGE_2_ (10^−6^ M/10^−7^ M/10^−8^ M); (v) ethanol (10^−6^ M/10^−7^ M/10^−8^ M) which served as vehicle control for PGE_2_ and corticosterone (vi) CM control without any drugs. After 24 or 48 hours of incubation, cells were collected from each well, counted and inspected for vitality using flow cytometry (see methods). Plates were then inspected under the microscope and no adhering cells were found. Under control conditions, CRNK-16 cells proliferated and increased their numbers approximately 3-fold per day. No differences were found between different control groups (v and vi) and therefore these groups were combined. The impacts of hormones and drugs were very similar at 24 (See Fig. S3 in Supporting Information) and 48 hours (Fig. 7); PGE_2_ and corticosterone decreased cell proliferation by approximately 50% (Fig. 7: A; p = 0.0241 and p = 0.0053 at 48 hrs, respectively) and cell vitality by approximately 10% (Fig. 7: B; p<0.0001 at 48 hrs), in a dose-dependent manner.

## Discussion

The present study clearly demonstrates that stressful conditions and physiologically relevant doses of stress hormones and prostaglandins can significantly increase mortality rates of leukemic rats. To the best of our knowledge, this is the first thorough investigation conducted in the realm of hematological tumors, specifically leukemia, causally demonstrating marked adverse effects of stress hormones and PGE_2_ on survival rates of leukemic animals. Specifically, our data showed that, in rats already bearing leukemia (for two or six days), exposure to a combination of epinephrine and corticosterone, for only several hours, significantly decreased survival rates. When these stress hormones were administered simultaneously with leukemia cells, an approximate 3-fold decrease in survival rates was evident. PGE_2_ and epinephrine by themselves caused similar effects, while corticosterone alone had smaller and non-significant effects. To test the impact of environmentally induced physiological stress responses, we employed a short paradigm of swim stress and found that the endogenous neuroendocrine responses markedly reduced survival rates of the CRNK-16 challenged rats. Furthermore, an acute administration of the β-blocker, nadolol, significantly attenuated this effect of stress, without affecting survival rates of non-stressed animals. Even in the absence of external stressful stimuli, tumor-associated or baseline levels of PGE_2_ and epinephrine contributed to the progression of CRNK-16 leukemia, as the prolonged administration of the β-blocker (nadolol) or of a COX inhibitor (indomethacin) significantly improved survival rates. Given that stress hormones were shown herein as having a wide window of opportunity to affect CRNK-16 progression (i.e. administered at 0, 2, and 6 days following tumor transfer), it seems expected that chronic exposure to the blockers (Exp. 4), but not acute exposure (Exp. 3), will increase survival rates that are modulated by the continuous tumor-associated or baseline levels of prostaglandins and catecholamines. In accordance with these findings, and using the same CRNK-16 model, we also recently reported that in leukemic rats undergoing surgery, which are clearly subjected to long-lasting high endogenous levels of catecholamines and prostaglandins, the combination of indomethacin and nadolol in a slow-release preparation significantly improved survival rates [2]. It is worthy to note, though, that in the current study we did not compare concentrations of nadolol in the blood between the acute and chronic administration procedures, and thus both the duration and the concentrations of the drug may underlie the difference between acute and chronic exposure. Overall, these and our previous findings demonstrate the deleterious impacts of endogenous stress hormones and PGE_2_ on survival of leukemia-bearing animals.

Immune suppression has long been suggested as a key mediator of the effects of stress responses on tumor progression. The sympathetic nervous system innervates all lymphoid organs [61], and lymphocytes (including NK cells and cytotoxic T cells) express receptors for catecholamines [62], [63], prostaglandins [64], [65], and glucocorticoids [66]. Stimulation of these receptors affects cytokine release [66], controls proliferation [67] and cell distribution [68], [69], [70], [71], and has repeatedly been shown to suppress many aspects of CMI, NK cell and CTL activity in particular [17], [48], [72], [73]. However, in order to relate reduced survival rates, as evident in the current study, to suppression of CMI, one needs to establish a role for CMI in controlling CRNK-16 progression. Indeed, we previously reported that the CRNK-16 line exhibits reduced expression levels of MHC-I [39], as is common in many human cancers including leukemia [74]. This common escape mechanism, efficient vis-à-vis adaptive immunity, facilitates detection and destruction of tumor cells by innate immunity, specifically NK cells [75], [76]. Moreover, we reported that the CRNK-16 tumor line is sensitive to NK-lysis *in vitro*, and that selective *in vivo* depletion of NK cells markedly decreased survival rates of CRNK-16 challenged rats [39]. Therefore, it can be concluded that stress-induced changes in NK activity are expected to modulate CRNK-16 progression. Hence, in the current study, we tested whether the observed effects of stress hormones and PGE_2_ on survival are associated with reduced NK activity. Indeed, our results indicated a significant decrease in NK activity against both the syngeneic CRNK-16 and the standard YAC-1 tumor lines following administration of epinephrine, corticosterone, or PGE_2_, and this reduction was mostly ascribed to reduced activity per NK cell. This suppression was evident at 90 minutes post hormone administration, but not two days later, as expected given the transient nature of the effects of these hormones on NK activity. We have previously shown that the swim stress paradigm used herein also suppresses NK activity [77] for a limited period of time [78]. These findings further strengthen (but do not prove) the proposed mediating role of CMI suppression in the effects of stress on leukemia progression.

These findings are in agreement with clinical findings indicating that the status (number and activity) of NK cells in leukemia patients is a predictor of prognosis [25], [26]. A potential role for NK cells in the rejection of leukemia is further suggested by the clinical efficacy of allogeneic bone marrow transplantation using non-identical HLA donors. Studies have shown [32] that implantations using selective mismatch in appropriate HLA loci (specifically NK KIR ligand) were more effective than matched combinations, enabling a graft-versus-leukemia activity due to the failure of recipient's leukemic cells to inhibit donor's NK cells through KIR receptors.

As expected, and given known *in vivo* interactions between epinephrine [79], PGE_2_ [80] and corticosterone, the administration of PGE_2_ and epinephrine in the current study transiently increased corticosterone serum levels. Surprisingly, the administration of a miniscule dose of 60 CRNK-16 cell transiently reduced corticosterone serum levels (at 90 minutes, but not at 48 hours) and increased NK activity levels against YAC-1 target cells. A potential mediator that may explain both findings could be a transient CRNK-16-induced secretion of IL-12, as IL-12 was reported to reduce corticosterone serum levels [81] and increase NK cytotoxicity [2].

As was discussed in the introduction, mechanisms other than immune modulation could mediate the effects of stress on tumor progression. In fact, several recent studies indicated that catecholamines can directly influence tumor cells, activating cellular pathways essential for their survival and growth. For example, the production of pro-angiogenic factors [24], [60], including VEGF, and of tumor invasion related enzymes, such as MMP2 and MMP9 [82], was upregulated *in vitro* by norepinephrine in several human and non-human cancers, through the β-adrenergic-cAMP-PKA pathway in tumor cells. In the current study and in our previous study [39], CRNK-16 bearing rats eventually developed disseminated tumors in various organs [38], which could then benefit from the release of such pro-angiogenic and invasion-promoting factors. In fact a role for VEGF was recently found in the *in vivo* progression of two animal leukemia lines [83]. Therefore, we tested whether CRNK-16 tumor cells produce VEGF and/or if this production is induced or augmented by compounds which we found here to reduce survival rates. We observed no spontaneous secretion of VEGF by CRNK-16, nor secretion following exposure to norepinephrine, the β-agonist metaproterenol, PGE_2_, or corticosterone. In contrast, other rodent tumor cells which we tested herein, all of which are solid tumors, did secrete VEGF spontaneously.

It was also reported that activation of β-adrenergic receptors [84] or of glucocorticoid receptors [85], [86] can sometimes directly induce tumor proliferation and increase tumor vitality, although there is also evidence to the contrary [87], [88], [89]. Thus, we studied the *in vitro* proliferation and vitality of the CRNK-16 line when incubated with the aforementioned compounds at a range of doses. In contrast to the prediction that these hormones would facilitate tumor progression, as may have been suggested by the outcomes of our *in vivo* survival studies, we found that corticosterone and PGE_2_ actually reduced in-vitro tumor cell proliferation and vitality (by approximately 50% and 10%, respectively), while epinephrine and metaproterenol had no effects. Overall, these findings reduce the likelihood of direct effects of stress hormones/PGE_2_ on CRNK-16 cells as a potential mediating mechanism of the *in vivo* effects of these hormones on survival rates, and strengthen the proposed role for CMI suppression. However, these findings do not negate other direct effects of stress hormones on CRNK-16 cells that might promote their survival, invasion capacity, or other angiogenic related processes in the more complex *in vivo* milieu.

Patients undergoing treatment for hematological malignancies experience high levels of anxiety and depression [33], [34], [35]. These two psychological factors were repeatedly linked to profound CMI suppression through the release of stress hormones [11], [90], [91], and were also associated with poor survival in leukemia patients [92]. In addition, NK activity has been reported to be compromised in patients with leukemia [93]. Therefore, our current results suggest the need for clinical studies testing the use of β-blockers, COX inhibitors, and approaches to limit cortisol levels, as potential interventions in leukemia patients aimed at restoring NK function and improving survival rates under stressful and apparently non-stressful conditions. COX inhibitors and β-blockers are used in numerous clinical settings with relatively minimal side effects, although contraindications may clearly occur under some conditions. Semi-selective COX-2 inhibitors may be advantageous, as they are believed to cause fewer adverse reactions than non-selective COX inhibitors [94], and non-selective β-blockers could prove superior to selective β1-blockers, as immunocytes express both β1 and β2 receptors [95]. Overall, this study underlines the importance of considering stress responses, initiated by internal or external stimuli, in the clinical setting of leukemia treatment, specifically addressing the potential beneficial effects of β-blockers and COX inhibitors.

## Supporting Information

Figure S1 **Effects of administration of epinephrine, corticosterone, PGE_2_ and CRNK-16 cells on corticosterone serum levels.** Ninety minutes (but not 48 hours) after their administration, epinephrine and PGE_2_, each significantly increased corticosterone serum levels. Administration of CRNK-16 cells significantly decreased corticosterone levels at 90 minutes, compared to vehicle controls. Data are presented as mean+SEM (n = 10/group/time point).
(TIF)

Figure S2 **Effects of administration of epinephrine, corticosterone, PGE_2_ and CRNK-16 cells on PGE_2_ serum levels.** Ninety minutes after its administration, PGE_2_ significantly increased PGE_2_ serum levels compared to vehicle controls to values beyond maximum detection levels, these values were calculated by extrapulation of standard curve. 48 hours after administration, levels were returned to baseline. Data are presented as mean+SEM (n = 10/group/time point).
(TIF)

Figure S3 ***In vitro*** **effects of stress hormones and PGE_2_ on CRNK-16 proliferation and vitality at 24 hours.** Corticosterone and PGE2 reduced CRNK-16 cell proliferation and vitality in a dose dependent manner at 24 h. Epinephrine and metaproterenol did not affect proliferation (A) and vitality (B) rates. Data are presented as mean+SEM. * indicates a significant difference from the control group. Drug concentrations ranged from 10^−8^ M to 10^−5^ M, see Exp. 7 for details.
(TIF)

Table S1 **Effects of administration of epinephrine, corticosterone, PGE_2_ and CRNK-16 cells on numbers of leukocyte subsets at 90 minutes.** Ninety minutes after their administration, epinephrine and PGE_2_ significantly reduced numbers of circulating lymphocytes, and specifically, T cells. PGE_2_ and injection of CRNK-16 cells also reduced numbers of circulating NKTs. * indicates a significant difference from the control group. Data are presented as mean (SEM).
(DOC)

Table S2 **Effects of administration of epinephrine, corticosterone, PGE_2_ and CRNK-16 cells on numbers of leukocyte subsets at 48 hours.** Fourty-eight hours after drug/tumor administration, no significant differences were found between the groups in numbers of circulating leukocyte subsets. Data are presented as mean (SEM).
(DOC)
