# Supplementary figures and images for: Do Stress Responses Promote Leukemia Progression? An Animal Study Suggesting a Role for Epinephrine and Prostaglandin-E2 through Reduced NK Activity
Source: PLoS One. 2011 Apr 29;6(4):e19246. doi: 10.1371/journal.pone.0019246 (PMC3084788; doi:10.1371/journal.pone.0019246)

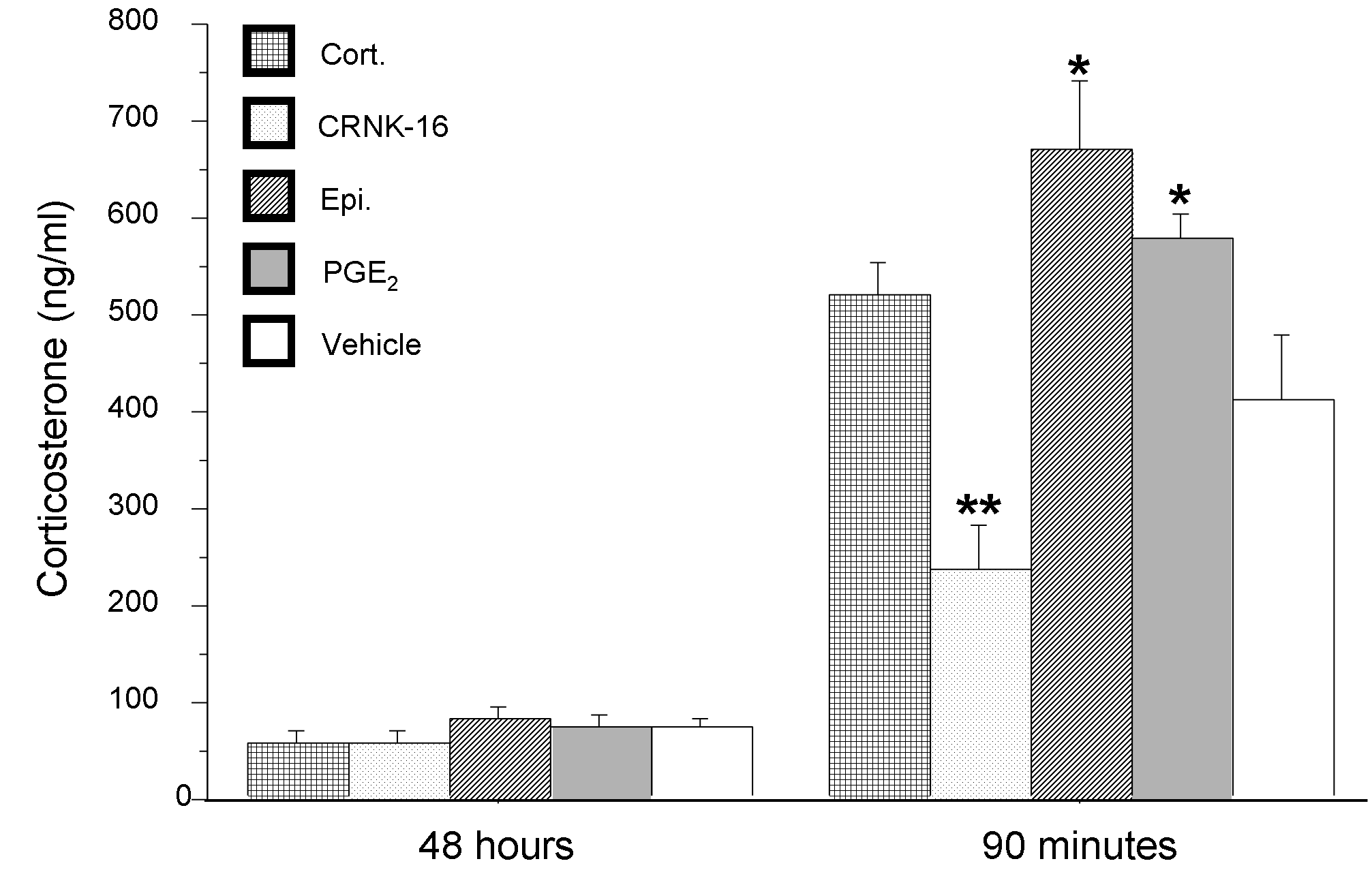

Supplement: Figure S1 — Effects of administration of epinephrine, corticosterone, PGE2 and CRNK-16 cells on corticosterone serum levels. Ninety minutes (but not 48 hours) after their administration, epinephrine and PGE2, each significantly increased corticosterone serum levels. Administration of CRNK-16 cells significantly decreased corticosterone levels at 90 minutes, compared to vehicle controls. Data are presented as mean+SEM (n = 10/group/time point). (TIF) [file pone.0019246.s001.tif]

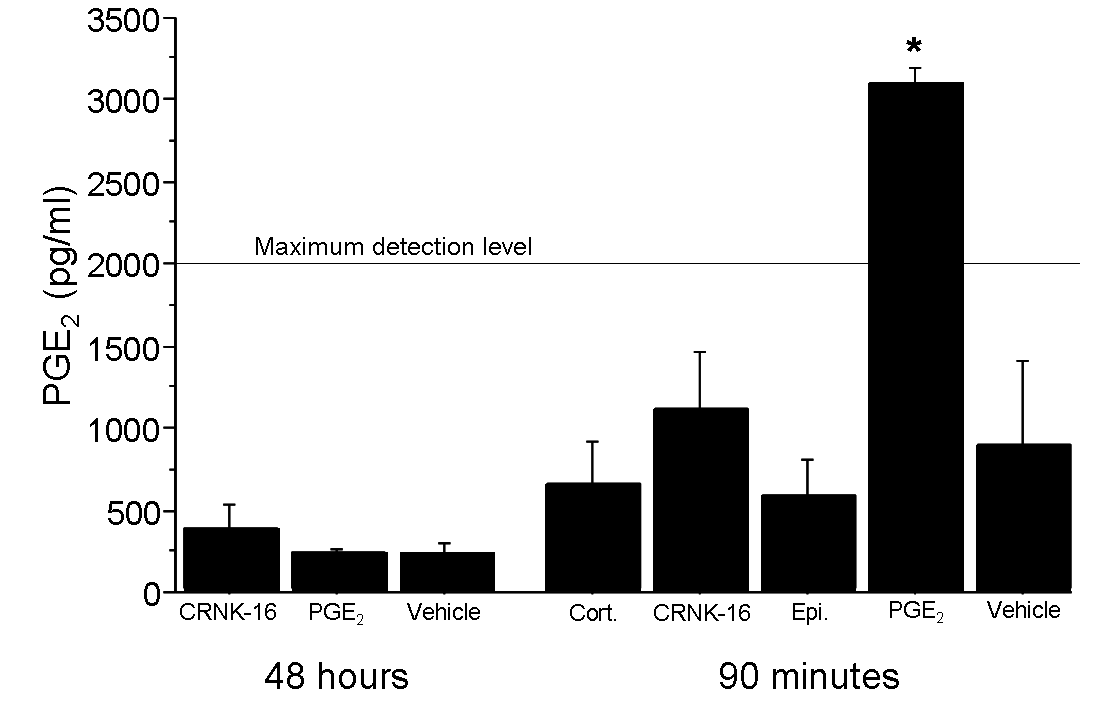

Supplement: Figure S2 — Effects of administration of epinephrine, corticosterone, PGE2 and CRNK-16 cells on PGE2 serum levels. Ninety minutes after its administration, PGE2 significantly increased PGE2 serum levels compared to vehicle controls to values beyond maximum detection levels, these values were calculated by extrapulation of standard curve. 48 hours after administration, levels were returned to baseline. Data are presented as mean+SEM (n = 10/group/time point). (TIF) [file pone.0019246.s002.tif]

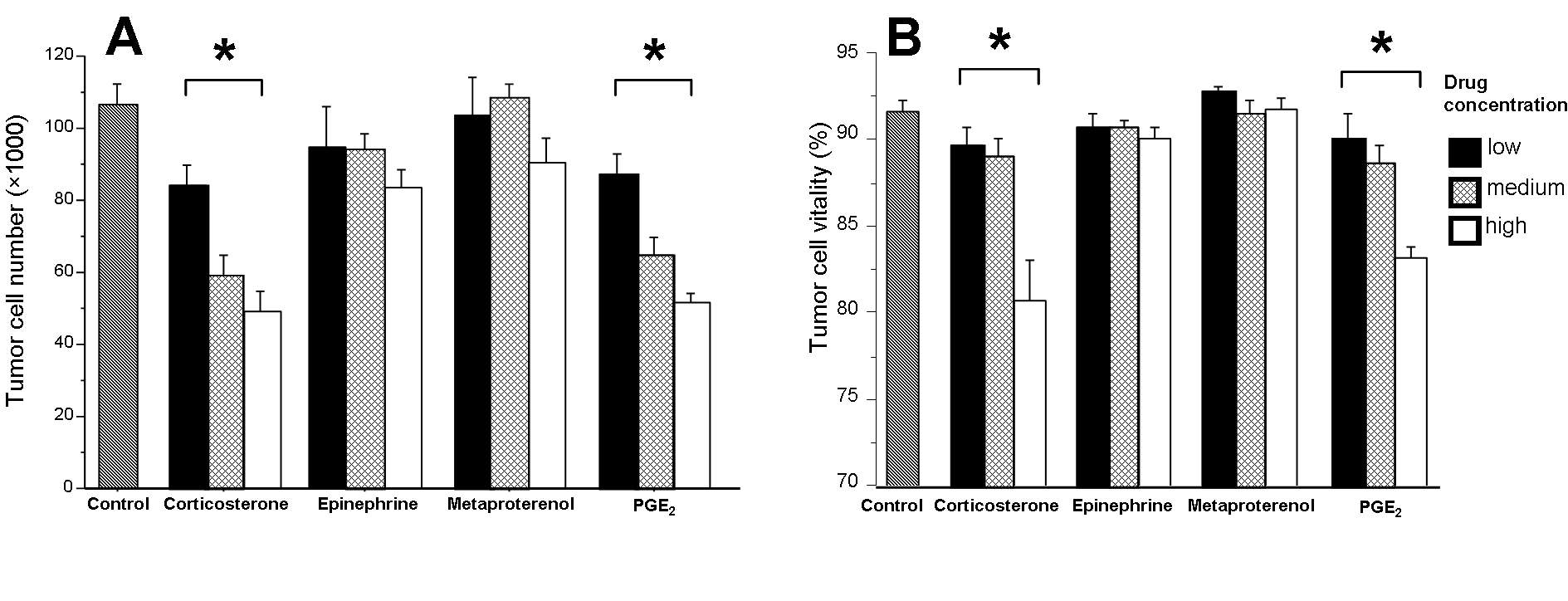

Supplement: Figure S3 — In vitro effects of stress hormones and PGE2 on CRNK-16 proliferation and vitality at 24 hours. Corticosterone and PGE2 reduced CRNK-16 cell proliferation and vitality in a dose dependent manner at 24 h. Epinephrine and metaproterenol did not affect proliferation (A) and vitality (B) rates. Data are presented as mean+SEM. * indicates a significant difference from the control group. Drug concentrations ranged from 10−8 M to 10−5 M, see Exp. 7 for details. (TIF) [file pone.0019246.s003.tif]
