# Supplementary material for: Do Stress Responses Promote Leukemia Progression? An Animal Study Suggesting a Role for Epinephrine and Prostaglandin-E2 through Reduced NK Activity
Source: PLoS One. 2011 Apr 29;6(4):e19246. doi: 10.1371/journal.pone.0019246 (PMC3084788; doi:10.1371/journal.pone.0019246)
Supplement: Table S1 — Effects of administration of epinephrine, corticosterone, PGE2 and CRNK-16 cells on numbers of leukocyte subsets at 90 minutes. Ninety minutes after their administration, epinephrine and PGE2 significantly reduced numbers of circulating lymphocytes, and specifically, T cells. PGE2 and injection of CRNK-16 cells also reduced numbers of circulating NKTs. * indicates a significant difference from the control group. Data are presented as mean (SEM). (DOC) [file pone.0019246.s004.doc]

**Table S1. Numbers of specific leukocyte subsets per microliter blood, 90 minutes following treatment**

|  | Granulocytes | Lymphocytes | T cells | NK cells | NKT cells |
| --- | --- | --- | --- | --- | --- |
| Vehicle | 1352.5 (176.8) | 3219 (293) | 2000.8 (184) | 175.4 (19.3) | 66 (7.6) |
| CRNK-16 | 1545.7 (111.4) | 3770 (187.7) | 2334.2 (110.5) | 177.8 (13.8) | 89 (8.1)* |
| Epinephrine | 1157.8 (172.2) | 2156.4 (103.9)* | 1386.9 (62.9)* | 201 (14.8) | 53.5 (6.1) |
| Corticosterone | 1459.8 (179.9) | 3258.2 (222.6) | 2075 (136.5) | 204.5 (20.3) | 63.1 (5.4) |
| PGE_2_ | 1500.6 (228.5) | 2379.9 (200.5)* | 1454.6 (111.8)* | 214.3 (27.3) | 36.4 (5.5)* |
