# Supplementary material for: Do Stress Responses Promote Leukemia Progression? An Animal Study Suggesting a Role for Epinephrine and Prostaglandin-E2 through Reduced NK Activity
Source: PLoS One. 2011 Apr 29;6(4):e19246. doi: 10.1371/journal.pone.0019246 (PMC3084788; doi:10.1371/journal.pone.0019246)
Supplement: Table S2 — Effects of administration of epinephrine, corticosterone, PGE2 and CRNK-16 cells on numbers of leukocyte subsets at 48 hours. Fourty-eight hours after drug/tumor administration, no significant differences were found between the groups in numbers of circulating leukocyte subsets. Data are presented as mean (SEM). (DOC) [file pone.0019246.s005.doc]

**Table S2. Numbers of specific leukocyte subsets per microliter blood, 48 hours** following treatment

|  | Granulocytes | Lymphocytes | T cells | NK cells | NKT cells |
| --- | --- | --- | --- | --- | --- |
| Vehicle | 1619.8 (221.1) | 5517.2 (261.3) | 2783.9 (152.8) | 187.8 (14) | 100.8 (10.3) |
| CRNK-16 | 1650.7 (238.9) | 5903.3 (368.9) | 3025.8 (160.4) | 189 (25.5) | 113.9 (10.2) |
| Epinephrine | 1614.1 (104.2) | 5271.2 (290) | 2740.5 (129.1) | 170.2 (15.2) | 95.7 (7.6) |
| Corticosterone | 1244.1 (102.3) | 5474.2 (371.8) | 2809.6 (209.5) | 172 (15.9) | 112.7 (16.9) |
| PGE_2_ | 1618 (232) | 4722.4 (265.1) | 2476.6 (122.9) | 170.2 (15.2) | 95.7 (7.6) |
